# Supplementary figures and images for: PDGFRα signaling regulates Srsf3 transcript binding to affect PI3K signaling and endosomal trafficking
Source: eLife. 2024 Dec 4;13:RP98531. doi: 10.7554/eLife.98531 (PMC11616996; doi:10.7554/eLife.98531)

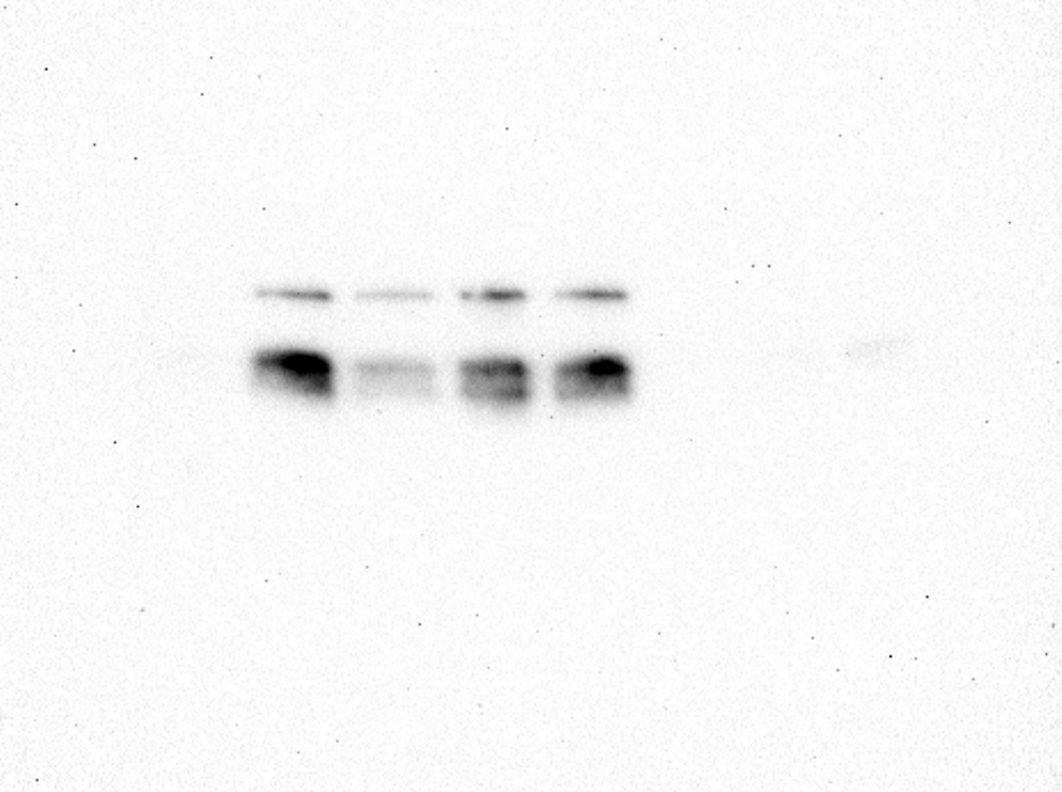

Supplement: Figure 1—source data 1. [file elife-98531-fig1-data1.zip › Figure 1 - source data 1/Figure 1 - source data 1 unlabeled.tif]

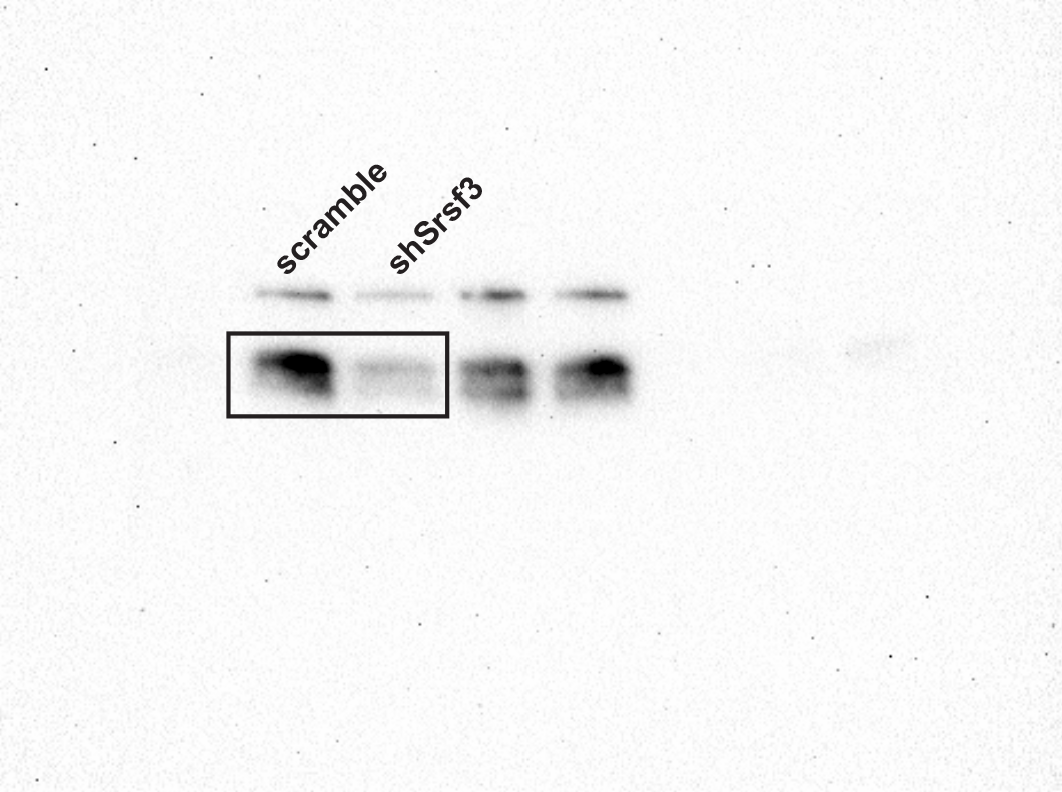

Supplement: Figure 1—source data 1. [file elife-98531-fig1-data1.zip › Figure 1 - source data 1/Figure 1 - source data 1 labeled.tif]

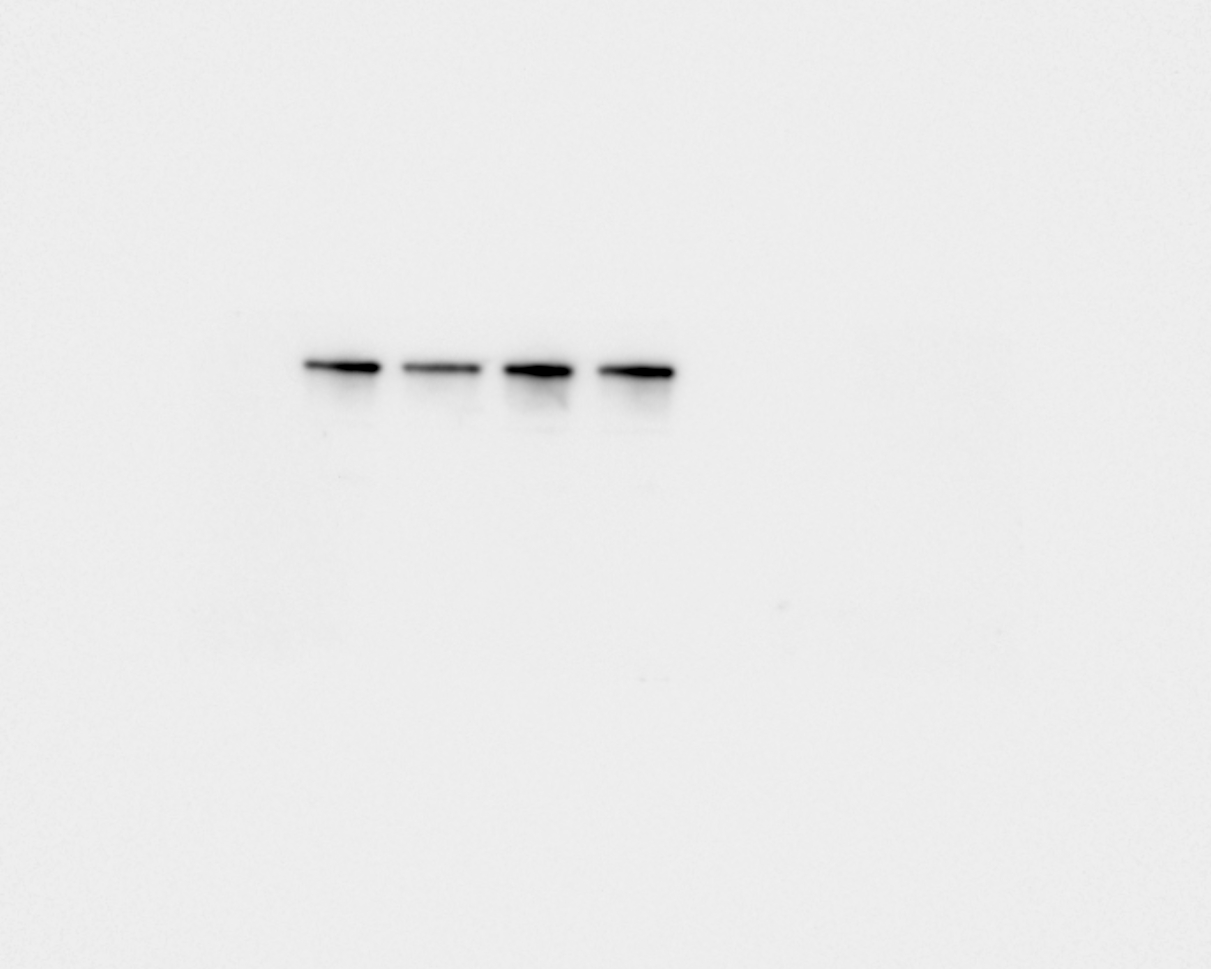

Supplement: Figure 1—source data 2. [file elife-98531-fig1-data2.zip › Figure 1 - source data 2/Figure 1 - source data 2 unlabeled.jpg]

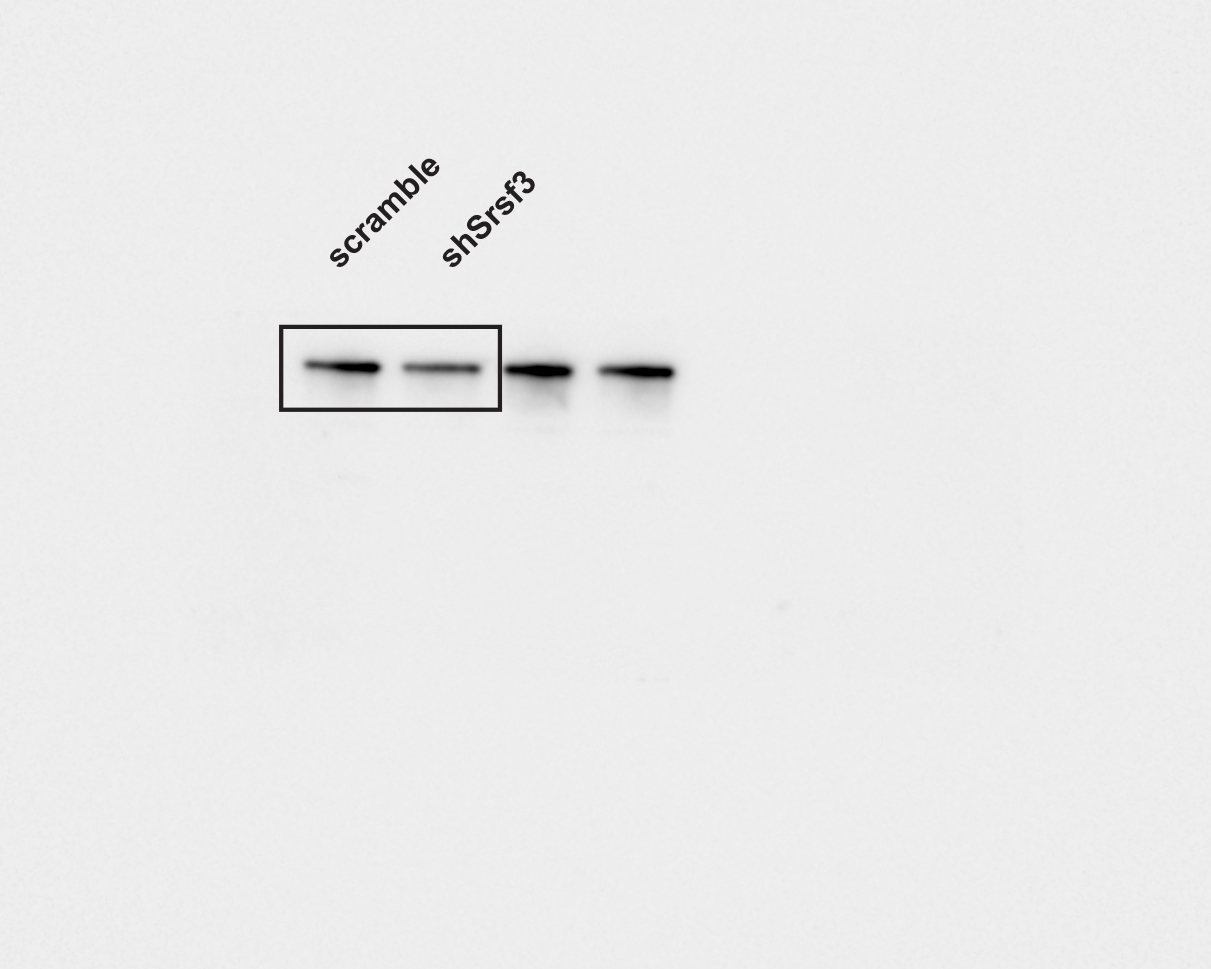

Supplement: Figure 1—source data 2. [file elife-98531-fig1-data2.zip › Figure 1 - source data 2/Figure 1 - source data 2 labeled.tif]

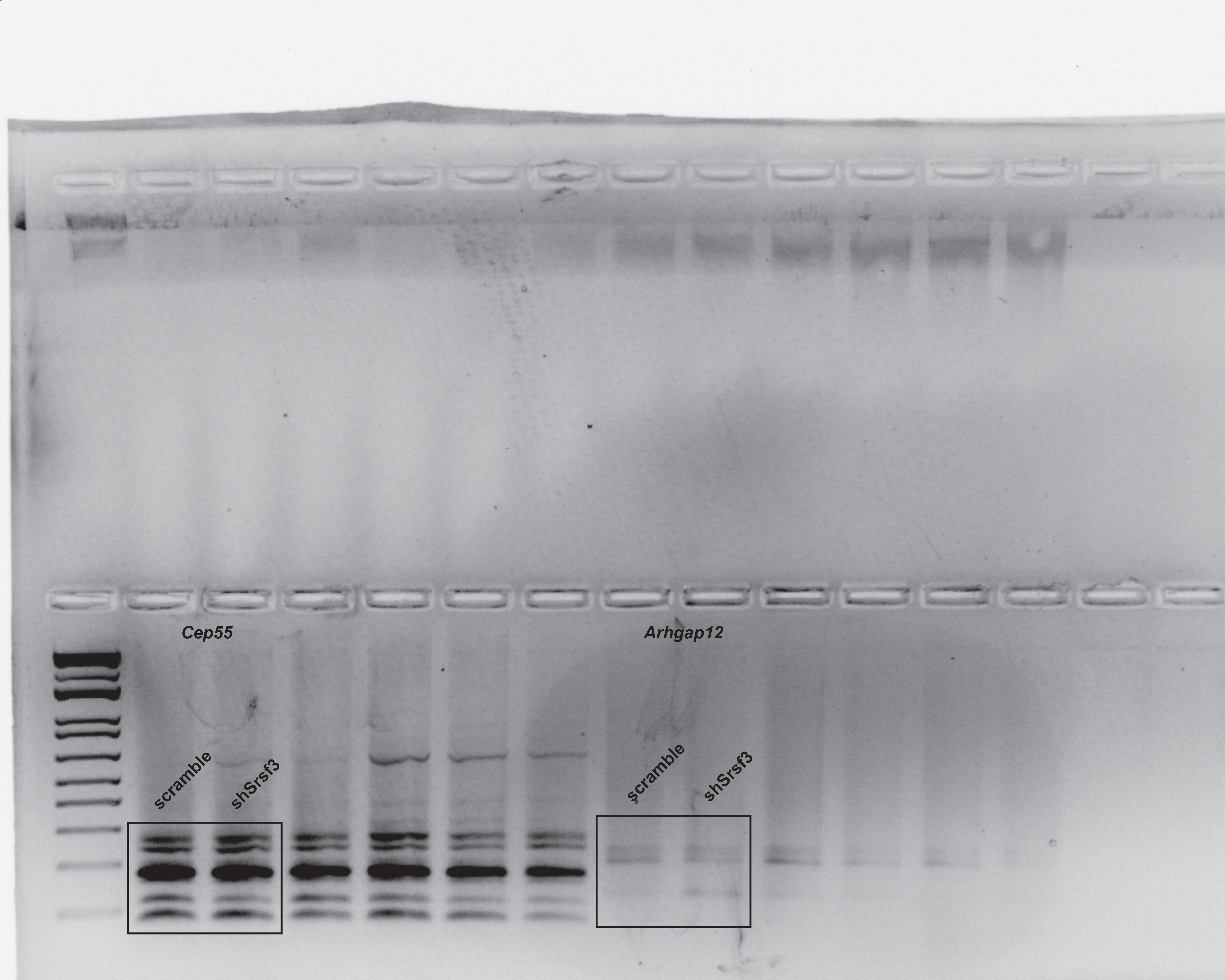

Supplement: Figure 2—figure supplement 1—source data 1. [file elife-98531-fig2-figsupp1-data1.zip › Figure 2 - figure supplement 1 - source data 1 labeled.tif]

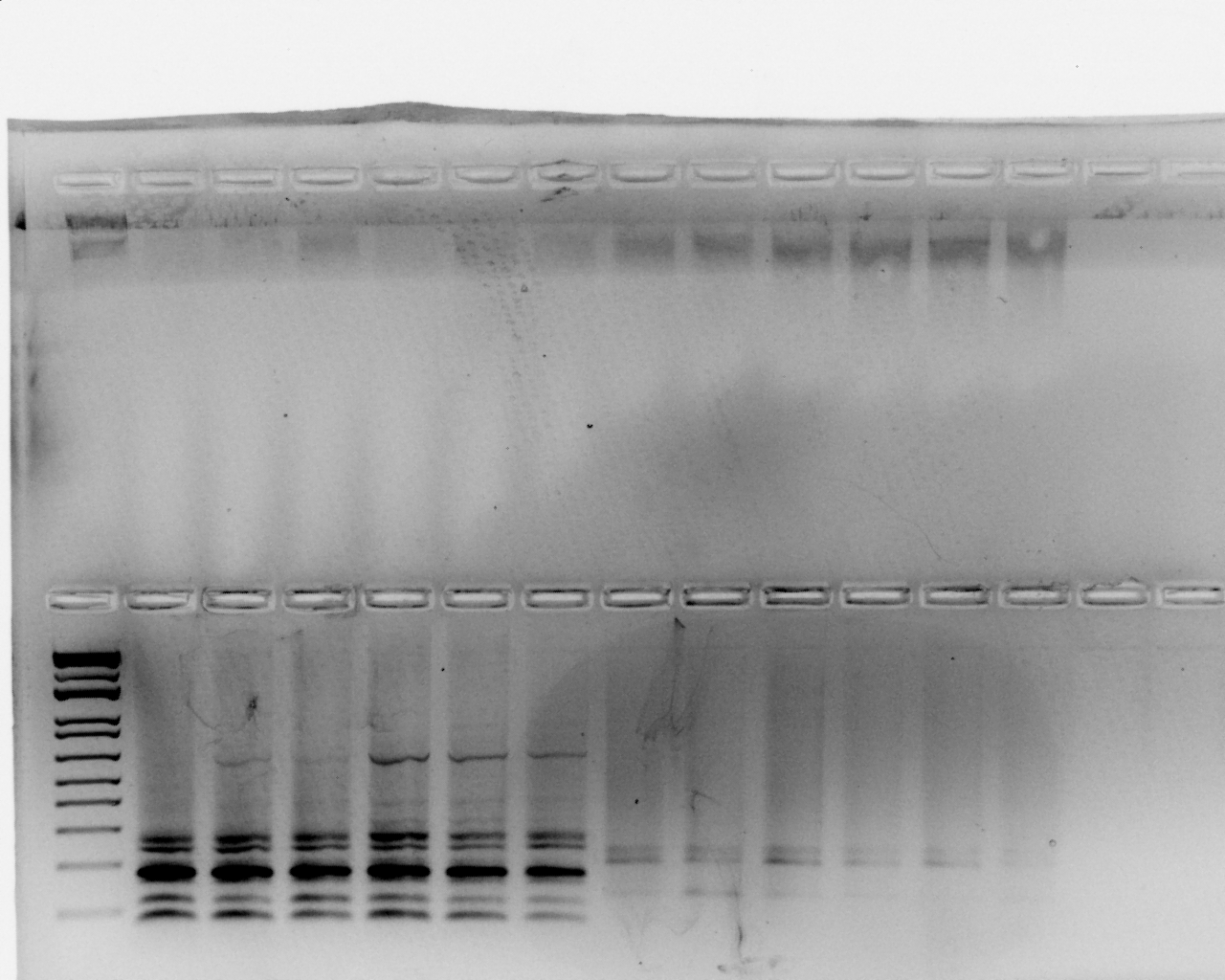

Supplement: Figure 2—figure supplement 1—source data 1. [file elife-98531-fig2-figsupp1-data1.zip › Figure 2 - figure supplement 1 - source data 1 unlabeled.tif]

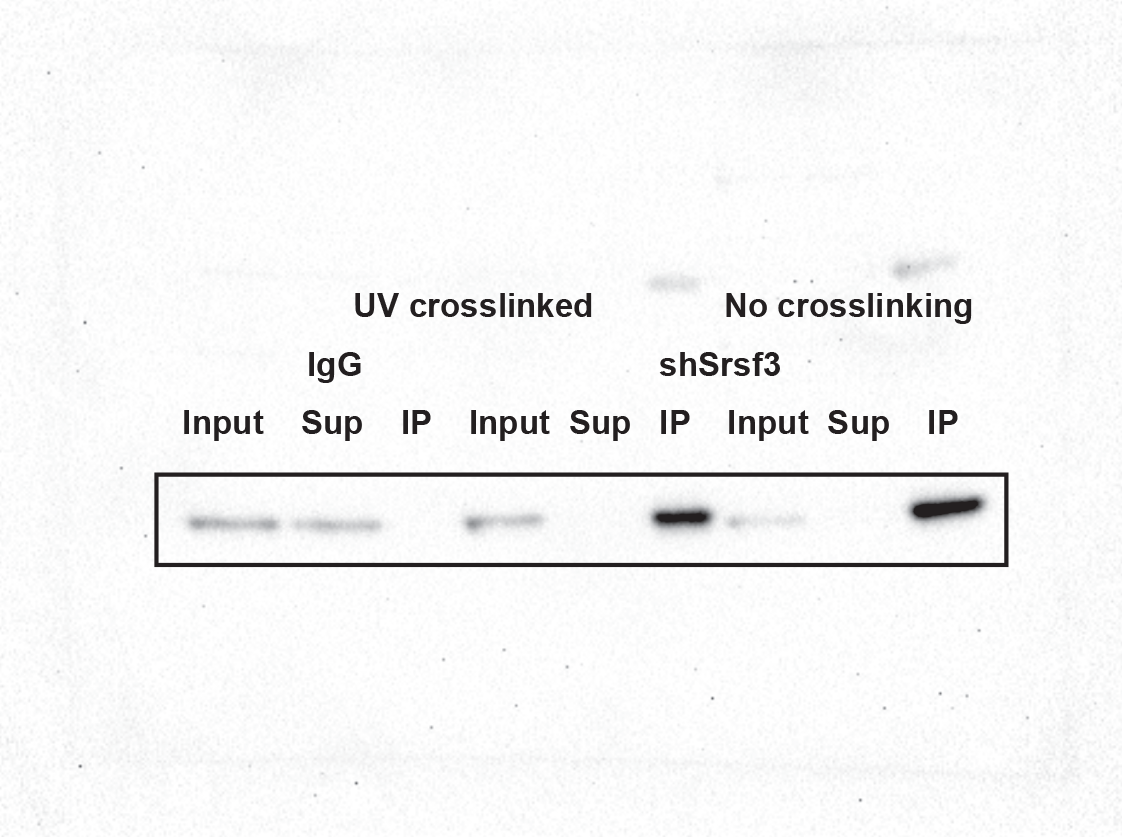

Supplement: Figure 3—source data 1. [file elife-98531-fig3-data1.zip › Figure 3 - source data 1/Figure 3 - source data 1 labeled.tif]

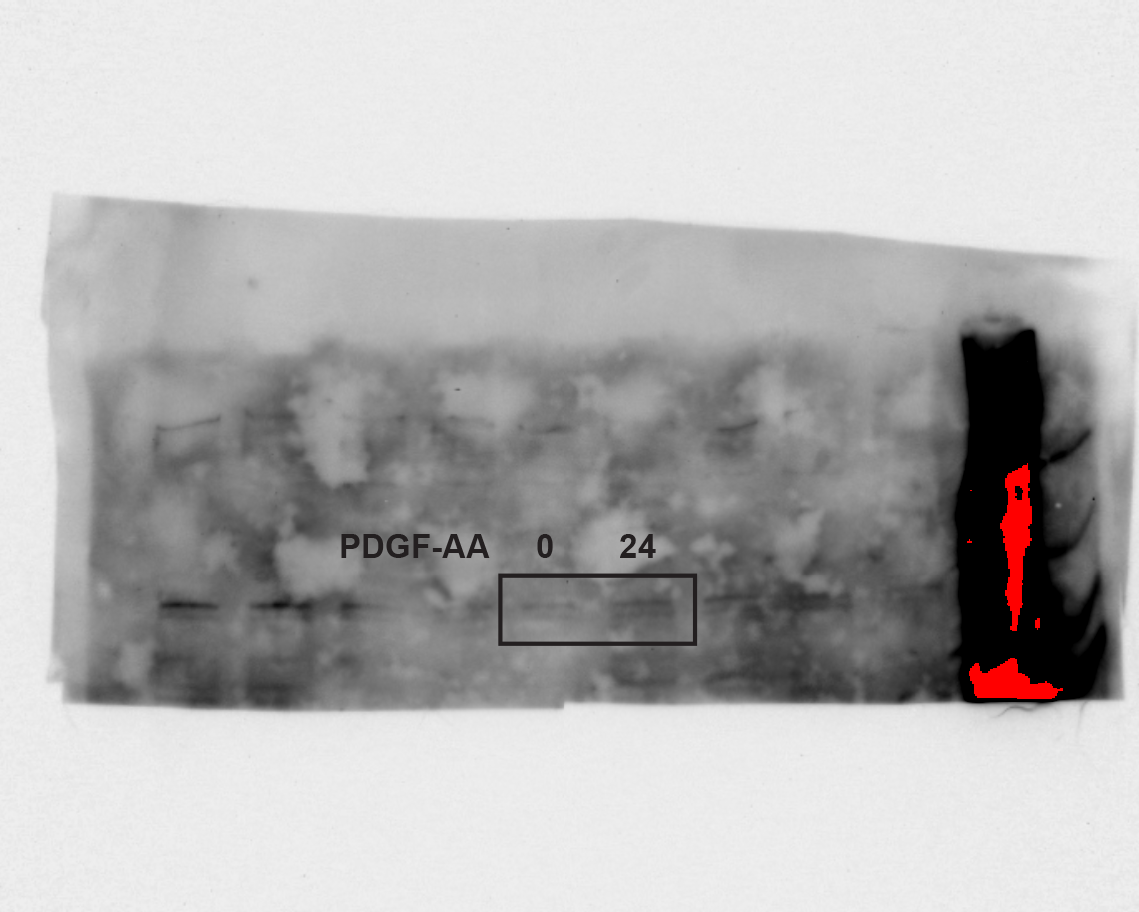

Supplement: Figure 5—source data 6. [file elife-98531-fig5-data6.zip › Figure 5 - source data 6/Figure 5 - source data 6 labeled.tif]

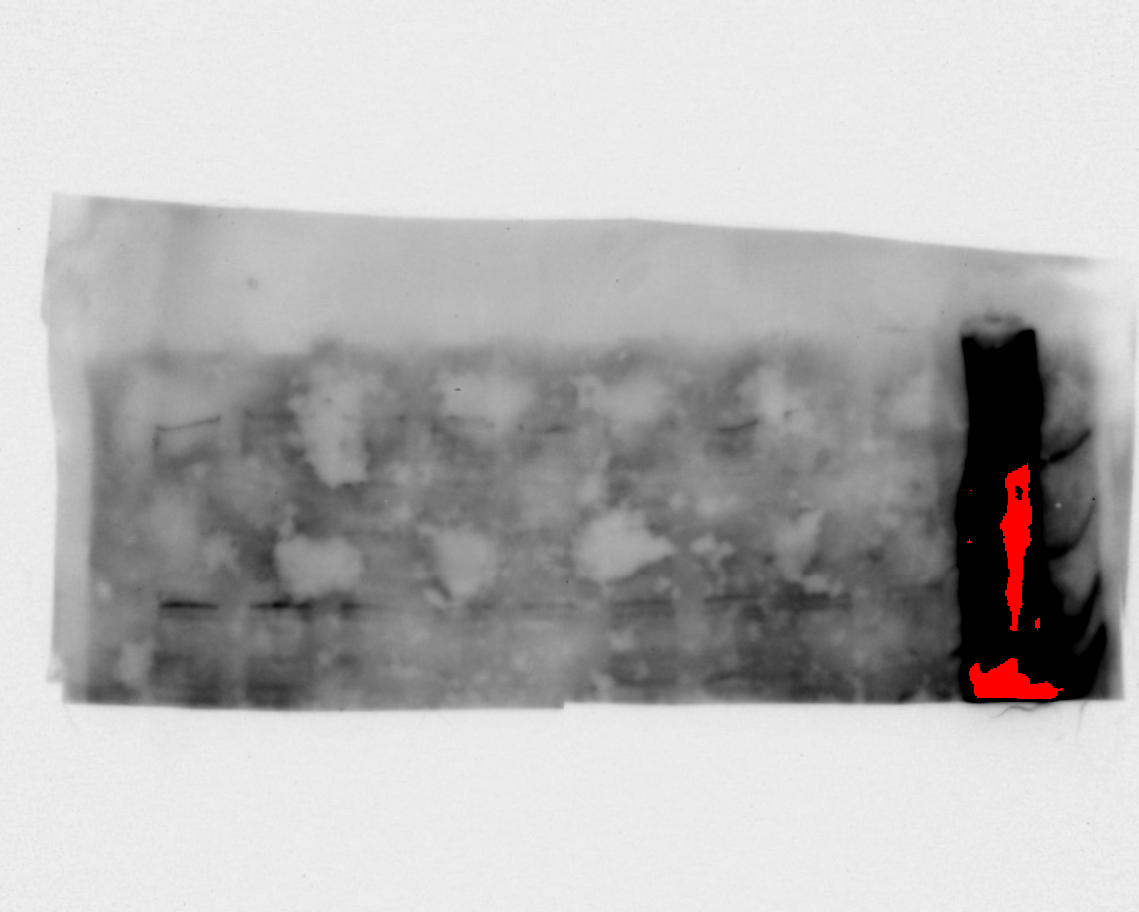

Supplement: Figure 5—source data 6. [file elife-98531-fig5-data6.zip › Figure 5 - source data 6/Figure 5 - source data 6 unlabeled.tif]

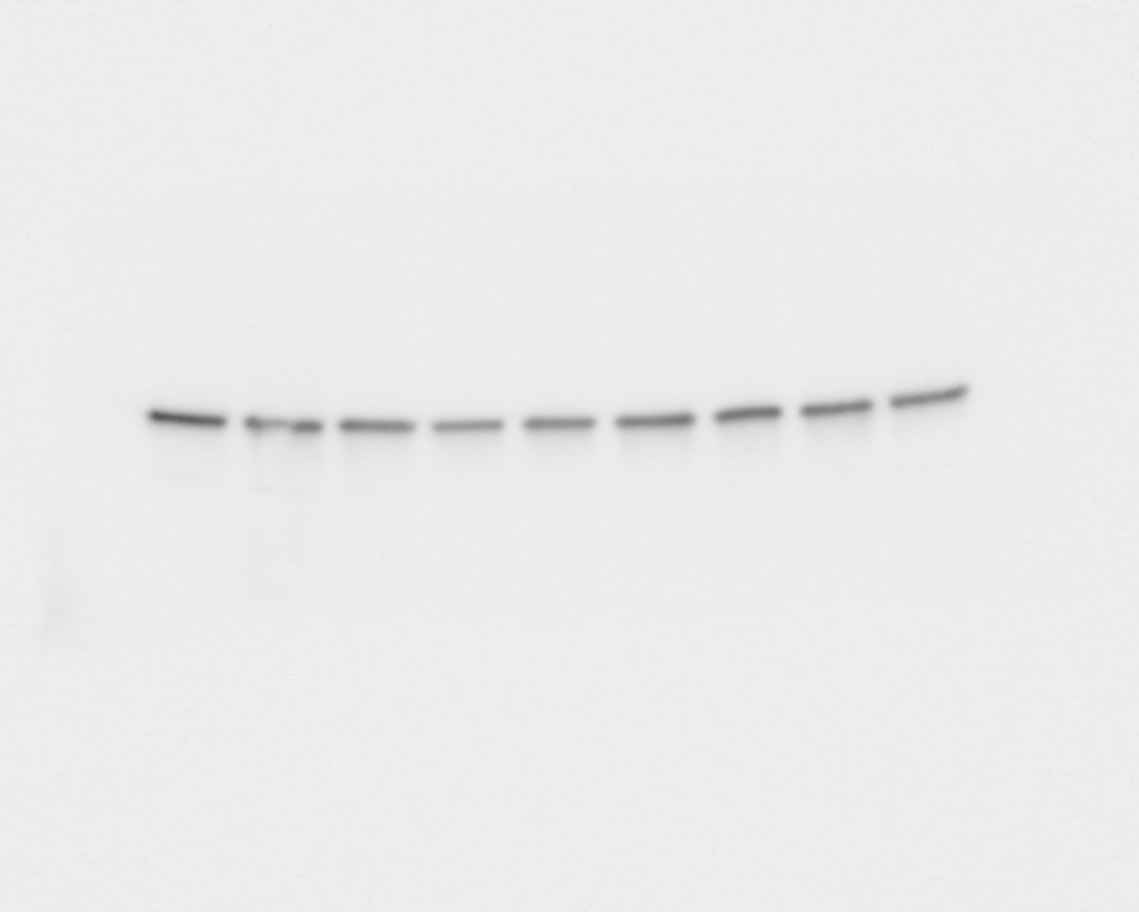

Supplement: Figure 5—source data 7. [file elife-98531-fig5-data7.zip › Figure 5 - source data 7/Figure 5 - source data 7 unlabeled.tif]

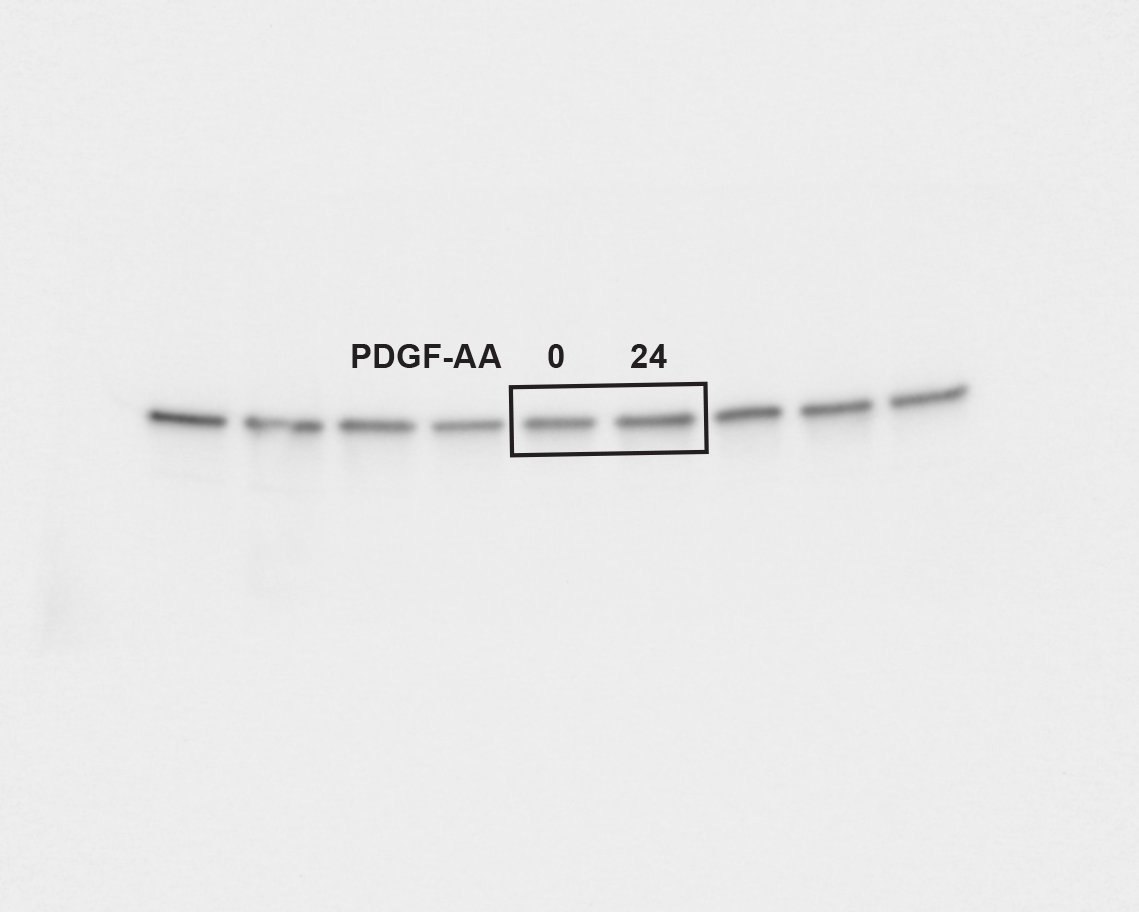

Supplement: Figure 5—source data 7. [file elife-98531-fig5-data7.zip › Figure 5 - source data 7/Figure 5 - source data 7 labeled.tif]

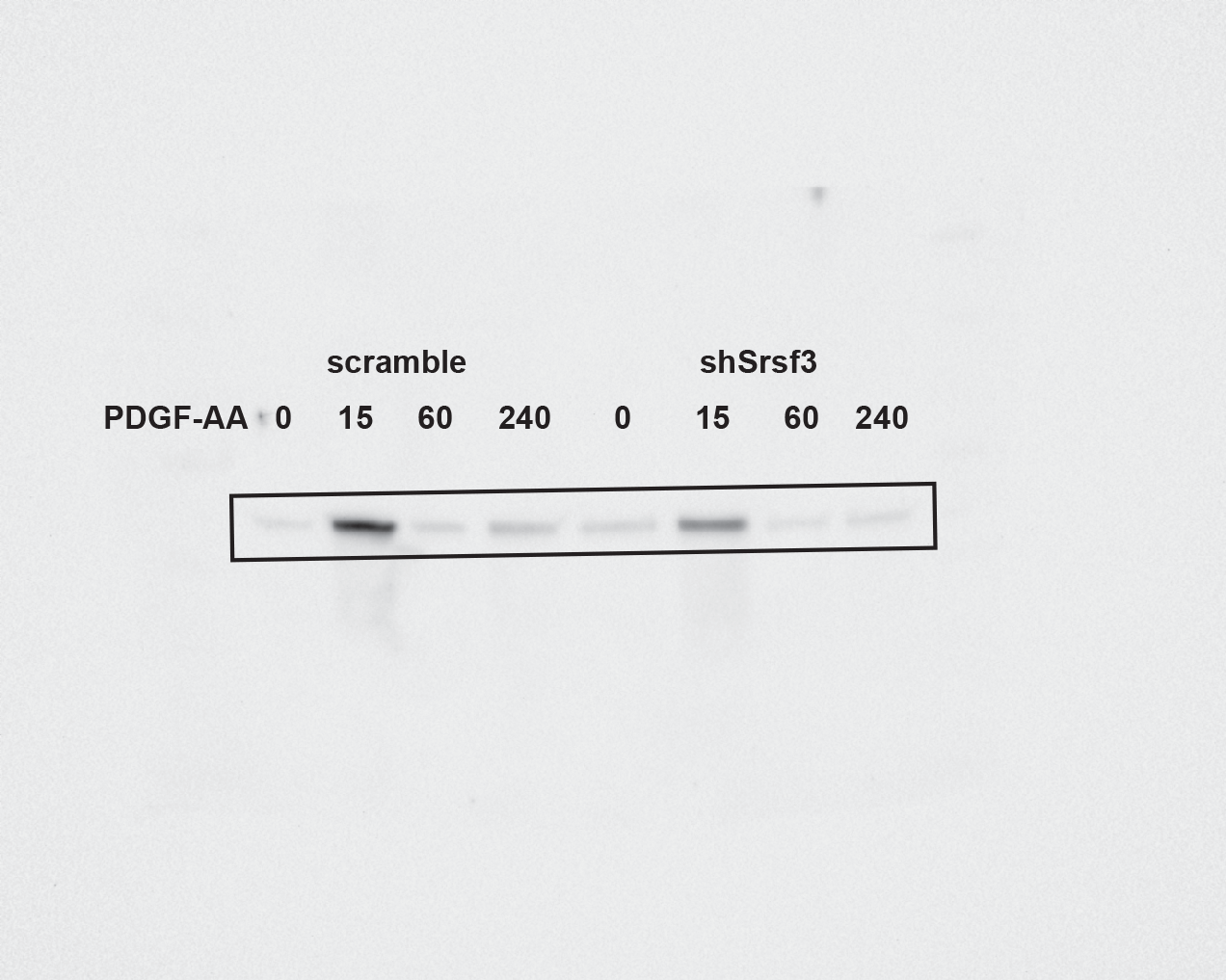

Supplement: Figure 6—source data 1. [file elife-98531-fig6-data1.zip › Figure 6 - source data 1/Figure 6 - source data 1 labeled.tif]

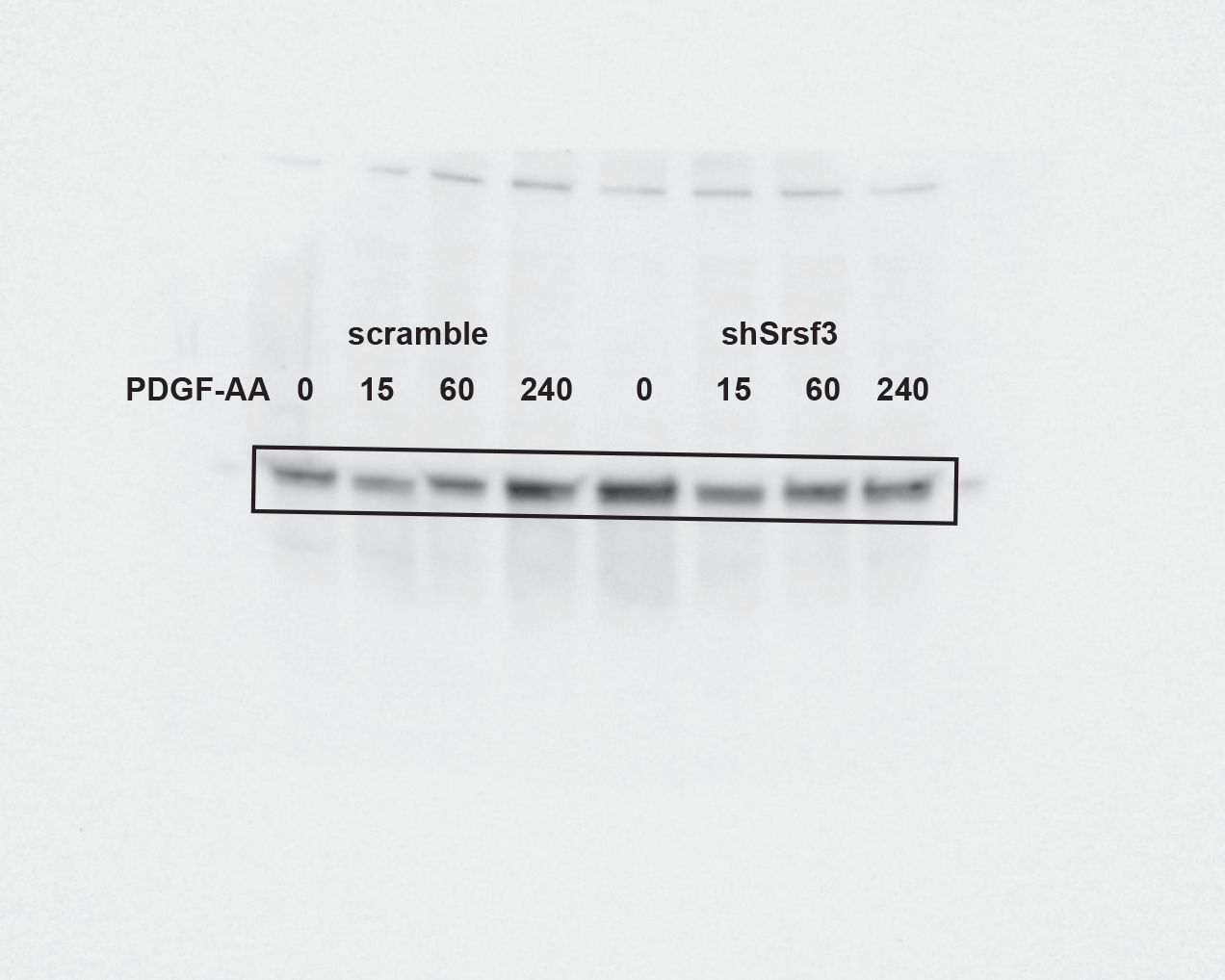

Supplement: Figure 6—source data 1. [file elife-98531-fig6-data1.zip › Figure 6 - source data 1/Figure 6 - source data 2 labeled.tif]

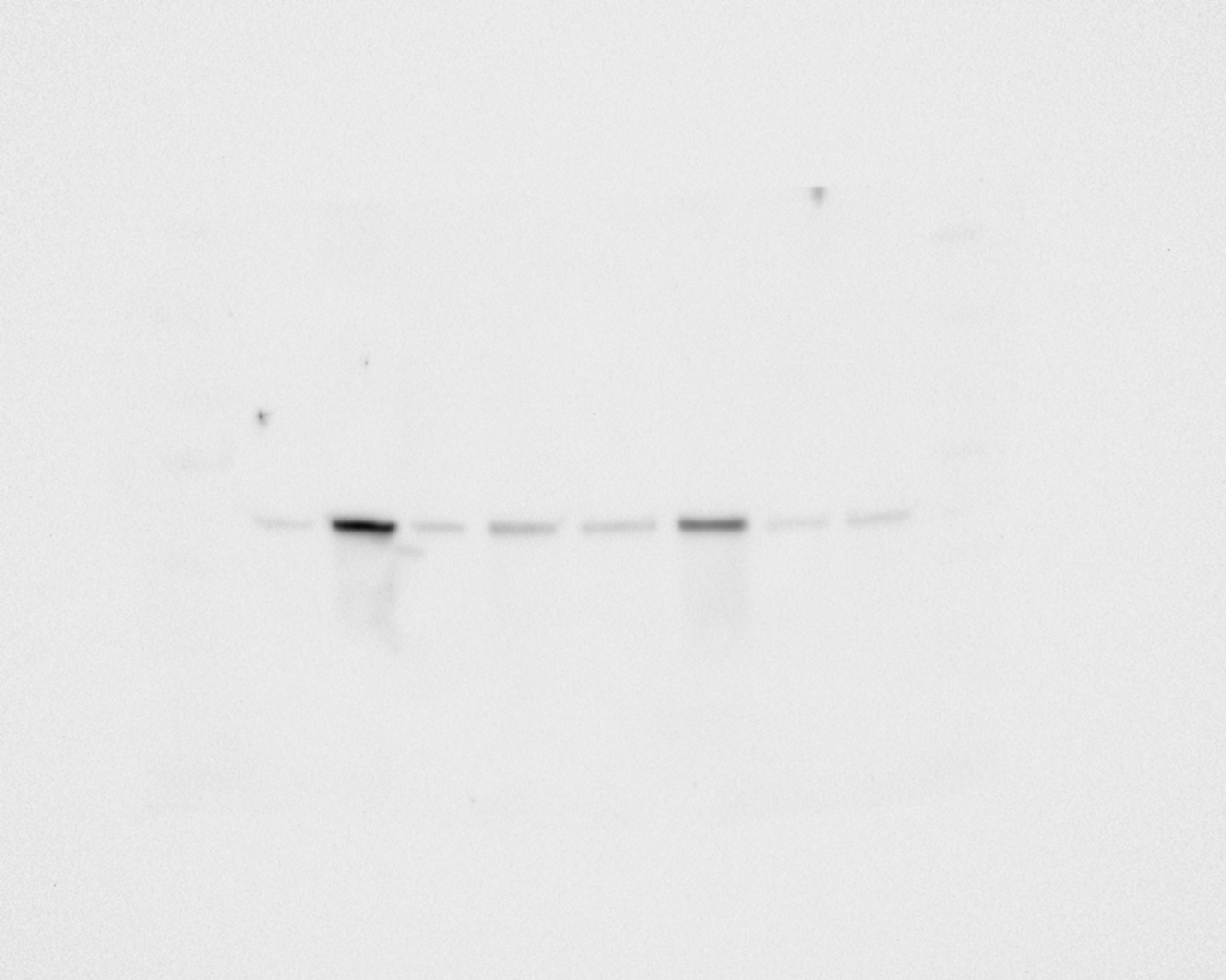

Supplement: Figure 6—source data 1. [file elife-98531-fig6-data1.zip › Figure 6 - source data 1/Figure 6 - source data 1 unlabeled.jpg]

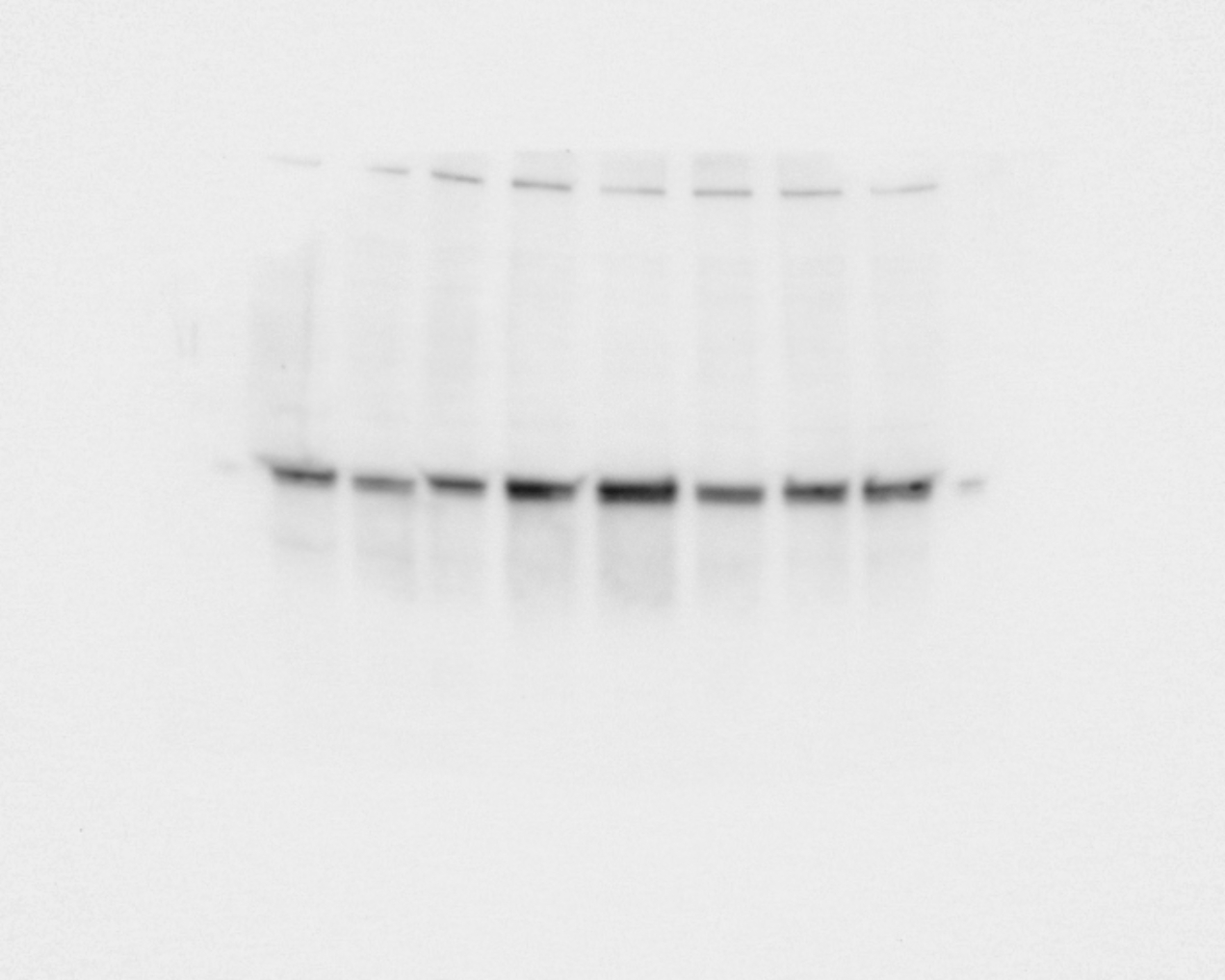

Supplement: Figure 6—source data 1. [file elife-98531-fig6-data1.zip › Figure 6 - source data 1/Figure 6 - source data 2 unlabeled.jpg]
